# The impact of death and dying on surgeons in a tertiary cancer centre in Singapore

**DOI:** 10.1186/s12893-025-02881-1

**Published:** 2025-05-05

**Authors:** Si Ying Tan, Jun Rong Tan, Yun Ting Ong, Yaoyi Ng, Annushkha Sinnathamby, Nur Amira Binte Abdul Hamid, Simon Yew Kuang Ong, Lalit Kumar Radha Krishna

**Affiliations:** 1 Division of Surgery & Surgical Oncology, National Cancer Centre Singapore, 30 Hospital Boulevard, Singapore, 168583 Singapore; 2 Yong Loo Lin School of Medicine, National University of Singapore, NUHS Tower Block, Level 11, Block 1E, Kent Ridge Road, Singapore, 119228 Singapore; 3 Division of Supportive and Palliative Care, National Cancer Centre Singapore, 30 Hospital Boulevard, Singapore, 168583 Singapore; 4 Division of Cancer Education, National Cancer Centre Singapore, 30 Hospital Boulevard, Singapore, 168583 Singapore; 5 Khoo Teck Puat National University Children’s Medical Institute, National University Health System, 5 Lower Kent Ridge Road, Singapore, 119074 Singapore; 6 Division of Supportive and Palliative Care, National University Cancer Institute Singapore, 5 Lower Kent Ridge Road, Singapore, 119074 Singapore; 7 Division of Medical Oncology, National Cancer Centre Singapore, 30 Hospital Boulevard, Singapore, 168583 Singapore; 8 Duke-NUS Medical School, National University of Singapore, 8 College Road, Singapore, 169857 Singapore; 9 Centre for Biomedical Ethics, National University of Singapore, Block MD11, 10 Medical Drive, #02- 03, Singapore, 117597 Singapore; 10 Palliative Care Institute Liverpool, Academic Palliative & End of Life Care Centre, Cancer Research Centre, University of Liverpool, 200 London Road, Liverpool, L3 9TA UK; 11 The Palliative Care Centre for Excellence in Research and Education, PalC, Dover Park Hospice, 10 Jalan Tan Tock Seng, Singapore, 308436 Singapore; 12 Health Data Science, University of Liverpool, Whelan Building, The Quadrangle, Brownlow Hill, Liverpool, Liverpool, L69 3GB UK

**Keywords:** Costs of caring, Compassion fatigue, Moral distress, Vicarious trauma, Secondary traumatic stress, Burnout, Death and dying, Surgeons, Surgery

## Abstract

**Background:**

Demands upon surgeons are increasing, especially in the care of the terminally ill. Fronting patient care, supporting families and guiding multi-disciplinary care teams facing the loss of patients see surgeons experiencing moral distress, compassion fatigue, vicarious trauma and secondary traumatic stress with the blurring of professional and personal boundaries. The full extent of these ‘costs of caring’ upon a surgeon remains unclear despite evidence of their compromises to patient care. To understand the costs of caring, semi-structured interviews are proposed to achieve the primary aim of understanding ‘What is known about the costs of caring on surgeons in Singapore?’ and the secondary aim, ‘What is the impact of the costs of caring on a surgeon’s professional identity?’.

**Methods:**

Twelve surgeons of various subspecialties from a tertiary cancer centre in Singapore were interviewed between 21st June 2022 and 18th December 2023. Transcripts were analysed using Krishna’s Systematic Evidence-Based Approach.

**Results:**

The key domains identified were: (1) motives for surgical career; (2) forms of emotional distress experienced; (3) impact of challenging experiences on personhood; and (4) buffer mechanisms.

**Conclusions:**

The costs of caring impact surgeons’ professional identities; shape their ‘internal compasses’ or the way they deliberate and assess their responsibilities; and influence patient safety and family support. The costs of caring and the ‘internal compass’ are moulded by the surgeon’s traits, maturing competencies, growing insights, clinical experience and sociocultural context. Without holistic, longitudinal and personalised support in both the personal and professional spheres, the costs of caring compromise surgeons’ confidence and professional identities.

**Supplementary Information:**

The online version contains supplementary material available at 10.1186/s12893-025-02881-1.

## Background

Surgical practice is evolving, none more so than in end-of-life care. Evolving social, legal and ethical expectations surrounding death and dying see surgeons leading conversations on care goals, consenting patients on sensitive end-of-life-care plans and supporting families, particularly in family-centric societies where family members play an active role in care [1–14]. Within Asian societies, surgeons also face unique ethically complex situations, such as collusion and familial determinations, which often come at the cost of the patient’s right to personal privacy, choice and self-determination [3–7, 9–12, 15–23].

These circumstances have been shown to lead to **moral distress** amongst surgeons, defined as the psychological disequilibrium rooted in the *“inability to act on core values and obligations”* [24]. Surgeons are also vulnerable to (a) **compassion fatigue**, a mix of physical and emotional exhaustion arising from prolonged and repeated engagement in emotionally demanding clinical care; (b) **vicarious trauma**, also referred to as ‘secondhand trauma’ from empathising deeply with patients’ suffering; (c) **secondary traumatic stress** that manifests as post-traumatic stress disorder (PTSD)-like symptoms from witnessing patients’ distressing situations or traumatic events; as well as (d) **burnout**, a persistent work-related physical and emotional exhaustion. This blend of interrelated concepts stemming from clinical care constitutes the costs of caring, a unifying framework that captures the complex interplay of emotional, social, psychological, ethical and professional demands in healthcare—offering a holistic understanding of how healthcare professionals, such as surgeons, are impacted by their caregiving professions. With the costs of caring associated with depression, anxiety, absenteeism, suicidal ideation and attrition and leading to diminishing surgical expertise in healthcare institutions [25–28], the need to enhance support for surgeons is imperative in safeguarding both sustainable, high-quality patient care and surgeon well-being. However, the full extent of the costs of caring upon surgeons remains unclear, including its nature, buffer mechanisms and the influence of both individual and sociocultural factors.

Guided by recent reviews into moral distress, compassion fatigue and the impact of caring for dying patients [28–36], we propose to study the costs of caring amongst surgeons in Singapore, where family-centric care is well-documented [13, 22]. In doing so, this study is envisioned to lay the groundwork for further studies to conceptualise and pilot support interventions relevant to the local practice setting, with the potential for regional adaptation and global expansion tailored to the needs of each country.

Hence, the aim of the research study is to address the primary research question, *‘What is known about the costs of caring on surgeons in Singapore?’*, and secondary question, *“What is the impact of the costs of caring on a surgeon’s professional identity?’*.

## Methods

### Theoretical lens: the ring theory of personhood (RToP)

We posit that the costs of caring for the dying, supporting their families and attending to individual distress of multi-disciplinary team members change a surgeon’s norms, beliefs, values, principles and roles (henceforth belief systems). Exposure to new belief systems and practices affects self-identity and self-concepts of personhood, or ‘what makes you, you’ [21, 37–39], that shape decisioning, actions and conduct. With such significance on practice and patient care, we utilise the Ring Theory of Personhood (RToP) [21, 37], a clinically evidenced theory of personhood. The RToP has been previously applied to similar studies on other populations and within the fields of medical education, ethics and palliative care [13, 21, 22, 28, 33–35, 40–42] to study shifts in belief systems. This theory was thus chosen for its ability to capture the fluid and context-dependent nature of personhood, making it particularly relevant for examining the evolving personal and professional identities of surgeons in response to their clinical experiences.

**Fig. 1:**
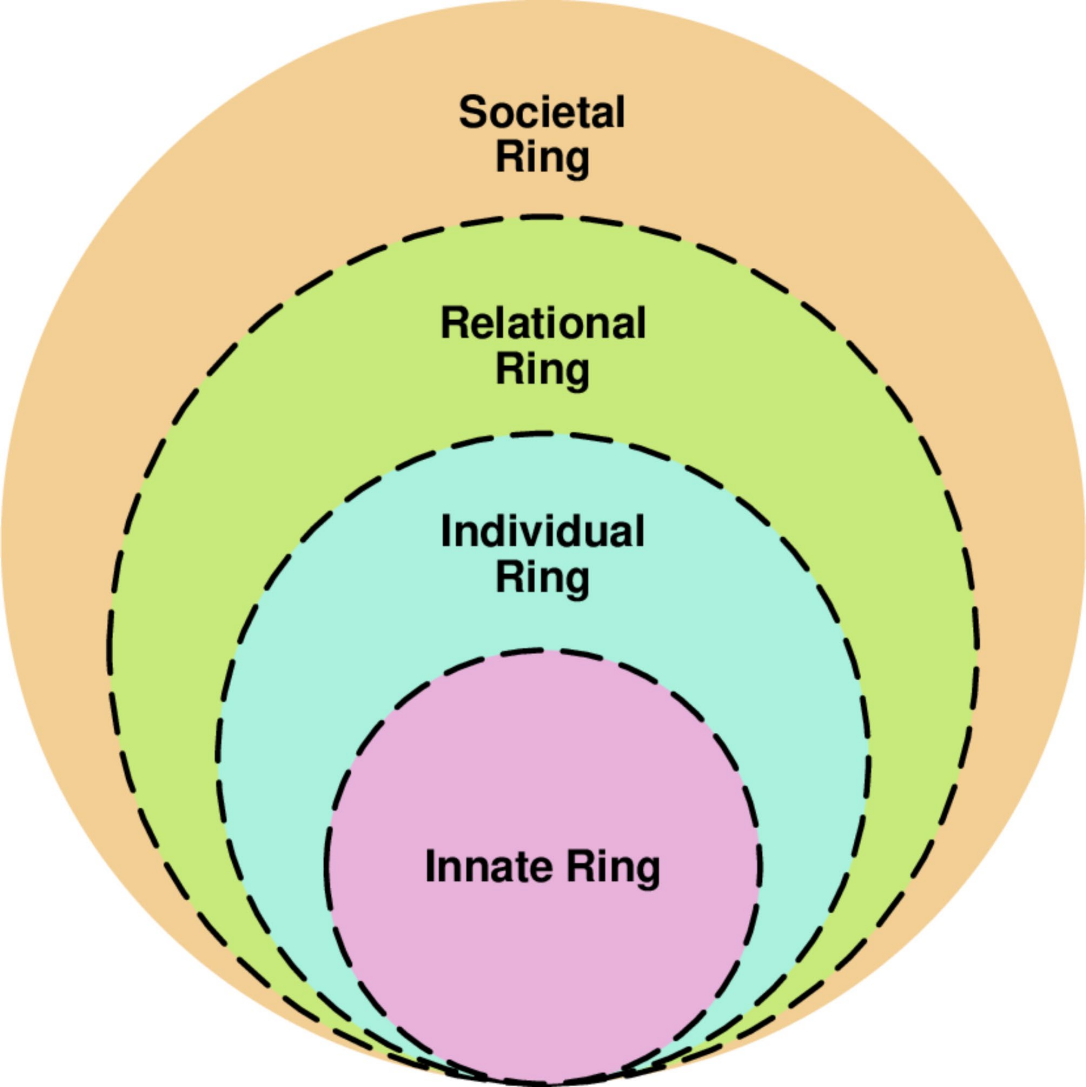
Ring theory of personhood [37]

The RToP maintains that there are four facets to belief systems, each corresponding to a domain of personhood. These are depicted by the Innate, Individual, Relational and Societal Rings (Fig. 1).

Revolving around the surgeon’s demographic traits, upbringing and religious and spiritual beliefs, the Innate Ring forms the core of what it means to be human. The belief systems guiding the surgeon’s conscious function, including behaviour, personality and values, comprise the Individual Ring. The Relational Ring focuses on intimate and personal relationships shared with family members and close friends whilst the Societal Ring is grounded in sociocultural, ethical, professional and legal norms, expectations, rights, roles and responsibilities.

**Fig. 2:**
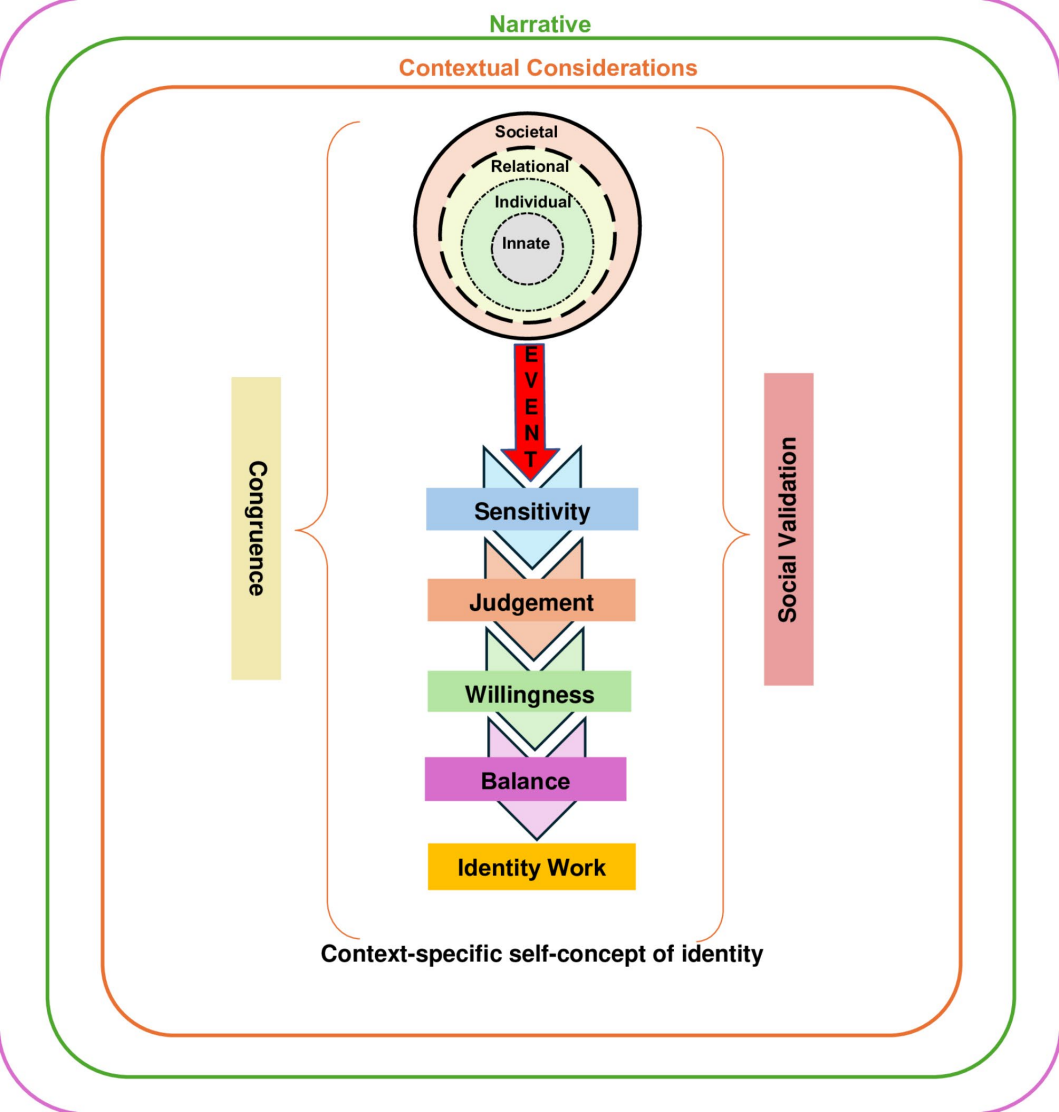
The Krishna-Pisupati Model for Professional Identity Formation. This model illustrates how events serve as catalysts for identity work and refinement of one’s professional identity

We believe that the costs of caring introduce new experiences, insights, beliefs and reflections that challenge current belief systems in one or more rings of the RToP. These challenges lead to shifts in self-concepts of personhood, which is then reflected in the surgeon’s self-identity. Such changes in belief systems influence their evolving sense of professional identity formation (PIF)—how they think, feel and act as professionals [43]. This is captured by the Krishna-Pisupati Model for Professional Identity Formation (KPM) (Fig. 2).

Figure 2 illustrates how surgeons’ professional identities may be conceptualised via the RToP. Through *events* that serve as catalysts for further reflection (e.g. the death of a patient), surgeons with *sensitivity* to such catalysts pass *judgement* regarding their significance and exercise a *willingness* to address and reflect upon such *events*. Subsequently, through careful *balancing* of pre-existing notions of self (i.e. their professional identity) and negotiation of *dissonance*, where new beliefs, experiences, insights and values are in conflict with pre-existing belief systems, surgeons practice *identity work* wherein they refine their professional identity to adapt to the larger sociocultural background and practice setting [44].

We posit that the twin lenses of the RToP and KPM will guide better appreciation and support of surgeons and facilitate institutional policies and educational programmes.

### The systematic evidence-based approach (SEBA)

To answer our primary research question, *‘What is known about the costs of caring on surgeons in Singapore?’*, and secondary question, *‘What is the impact of the costs of caring on a surgeon’s professional identity?’*, the research team drew upon Krishna’s Systematic Evidence-Based Approach (SEBA) [36, 45–54] and carried out semi-structured interviews of surgeons at a tertiary hospital in Singapore. Semi-structured interviews were chosen as the research methodology due to their flexibility in accommodating unique individual accounts to facilitate further exploration of ideas.

**Fig. 3:**
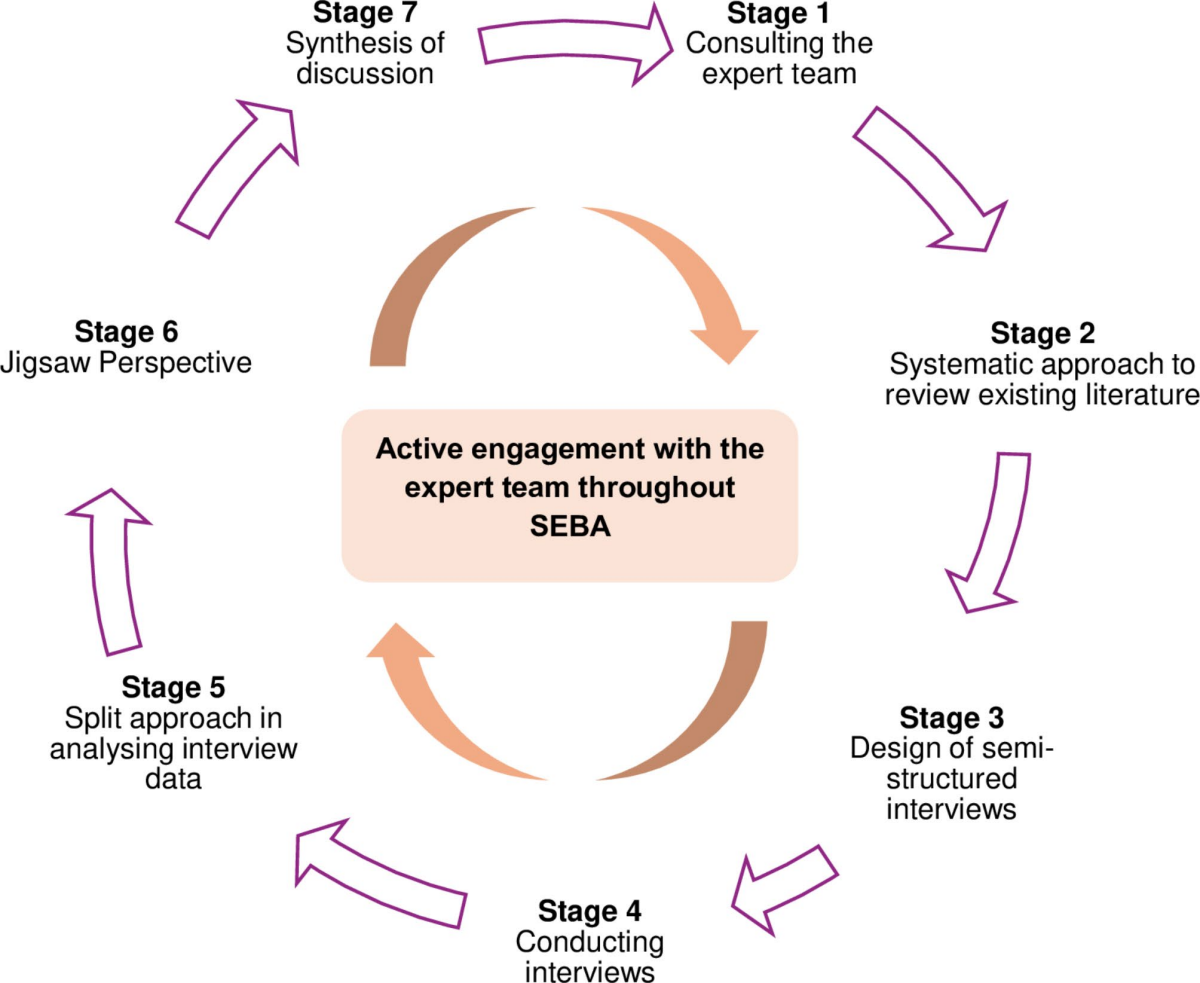
Systematic evidence-based approach (SEBA) process

Concurrently, SEBA’s constructivist and ontological lens captures the wider sociocultural influences on individual personhood and accommodates the use of the RToP and KPM to guide our analysis of semi-structured interview data [55, 56]. The stages of SEBA are delineated in Fig. 3 below.

### Ethics approval and consent to participate

Ethics approval (reference number: 2021/2176) was obtained from the SingHealth Combined Institutional Review Board. Informed written and oral consent was obtained from all participants.

#### Stage 1. expert team

The research team called upon an expert team of clinicians, clinician-educators and a medical librarian from an oncology centre, palliative care institute and local medical schools to serve an advisory role and provide oversight in the research process.

##### Reflexivity

The lead author was a senior breast surgeon with significant experience in undergraduate and postgraduate surgical education. Whilst her ‘insider’ role within the study offered nuanced understanding of the surgical training and practice structure and culture unknown to ‘outsiders’, potential drawbacks were imminent. These included biases in data analysis, which could lead to confirmation biases, alongside power imbalances between the lead author and study participants, potentially reducing the participants’ willingness to share negative accounts. To minimise these effects, the research team consisted of research assistants and medical students who were members of a peer-mentorship research programme, guided by palliative care consultants with expertise in medical education, qualitative analysis and conducting systematic reviews. By having research and expert teams engage in personal and group reflexivity, with a balance of ‘insider’ and ‘outsider’ perspectives throughout the research process, personal biases were identified and minimised through ongoing discussions. Additionally, research assistants who did not have a dependent relationship with the participants were recruited as interviewers, thereby mitigating social desirability bias. Further, this study adheres to the Consolidated Criteria for Reporting Qualitative Studies (COREQ) guidelines to ensure comprehensive and transparent reporting of the research process (see Additional File 1).

#### Stage 2. systematic approach

Guided by a systematic approach, the expert and research teams reviewed current accounts of the impact of death and dying on physicians to inform the design of a semi-structured interview guide.

#### Stage 3. design of semi-structured interviews

Seeking to capture how the personal beliefs, values and practices of surgeons were impacted by their lived experiences, local palliative care and oncology physicians and qualitative researchers reviewed and revised the interview guide (see Additional File 2) through a modified Delphi process [57].

#### Stage 4. conducting semi-structured interviews

Purposive sampling was used to select participants for the study. In 2022, 44 eligible surgeons from the department of surgery at a local tertiary cancer centre were sent email invitations to participate in the study, of which 12 physicians were recruited. The remaining 32 eligible surgeons did not respond or provide a reason for non-participation. Participants were informed of the study details, including its nature, duration and aims. Voluntary participation, participant anonymity and the right to withdraw at any point were stressed.

Before the commencement of the interviews between 21st June 2022 and 18th December 2023, informed written and verbal consent were obtained from all participants. The interviews were then performed independently by three trained female research assistants, M.C, A.S.I.L and N.A.B.A.H., with a master’s degree in medical humanities and a bachelor’s degree in psychology and sociology respectively. Each interviewer was also trained in theoretical concepts of RToP and KPM to elucidate subtle nuances and effective prompts for more robust interviews. Interview questions were reviewed with the interviewers to ensure a consistent understanding of the questions and concepts. Pilot interviews were also performed with the research team to confirm that the interviews were sufficiently in-depth and relevant to addressing the research questions. Further, the specific recruitment of these interviewers served to reduce the risk of social desirability bias as they did not share any prior or dependent relationship with the participants.

Held on a one-on-one basis with only the participant and one interviewer present, the interviews took place on an institutionally secured Zoom video-conferencing platform. Conducted in English, the interviews were audio-recorded, each lasting approximately an hour. Data collection and analysis occurred simultaneously. No field notes were made during or after the interviews. Recruitment and interviews ceased when no new insights or themes emerged from two consecutive interviews, with data saturation reached after nine interviews. No repeat interviews were performed. Each anonymised audio recording was transcribed verbatim and sent to each participant for member checking to ensure the accuracy of their statements.

#### Stage 5. split approach

The Split Approach involved the concurrent application of Braun and Clarke’s [58] thematic analysis and Hsieh and Shannon’s [59] directed content analysis by two sub-teams of researchers. This combined approach facilitated a review of the data from various viewpoints whilst safeguarding rigour and trustworthiness.

In applying Braun and Clarke’s [58] thematic analysis, the first team of two researchers (J.R.T. and Y.T.O.) read the anonymised transcripts to delineate striking patterns in the data. Codes were derived based on the ‘surface meaning’ of the transcripts, referring to their direct or literal interpretations. In an iterative step-by-step analysis, complementary codes were merged to synthesise sub-themes that were *“defined from the raw data without any predetermined classification”*. This process was repeated to formulate bigger themes from sub-themes.

Concurrently, Hsieh and Shannon’s [59] directed content analysis was performed by the second team of two researchers (A.S. and L.K.R.K.). This method utilised a template of pre-existing codes, drawn from Somasundaram et al.’s [28] study on the impact of death and dying on medical oncologists in Singapore, to analyse the data. New codes were assigned to any data incompatible with the pre-existing codes. Similarly, complementary codes were combined to form categories.

An exchange of broader patterns of meaning was shared between members within each team and an audit trail was maintained, using Microsoft Excel to manage the data. Consensus between members was achieved by practicing Sandelowski and Barroso’s [60] approach to *“negotiated consensual validation”*. All results were also discussed with the senior authors, T.S.Y., S.O.Y.K. and L.K.R.K. Themes and categories elucidated from the analysis were deemed to be reliable as independent researchers were in concordance. A reflexive approach was also applied with members reflecting upon their own biases individually and as a group to minimise bias and improve the validity of results.

#### Stage 6. jigsaw perspective

The Jigsaw Perspective entailed the melding of similar and/or overlapping categories and themes, akin to pieces of a jigsaw puzzle, to form a complete picture of the data. This process was guided by Phases 4 to 6 of France et al.’s [61] adaptation of Noblit and Hare’s [62] seven phases of meta-ethnography that prompted reciprocal translation of the complementary themes and categories such that they accurately reflected one another. To do so, the research team reviewed the codes and subcategories and/or subthemes.

## Results

The demographics of 12 participating surgeons may be found in Table 1 below.

**Table 1:** Demographics of participants, including years of practice and subspecialty

Surgeon	Years of Practice	Subspecialty (if mentioned)
S1	19 (as a surgeon)	Breast
S2	25 (as a surgeon)	Breast
S3	4	NA
S4	8 (in surgical oncology)	Surgical Oncology
S5	NA	Acute Care Surgery
S6	11	Gynae-Oncology
S7	4 (as a breast surgeon)	Breast
S8	10	Breast
S9	10	NA
S10	18	NA, but patient profile is generally those with late-stage cancers
S11	23	NA
S12	5	Head and Neck

**Fig. 4:**
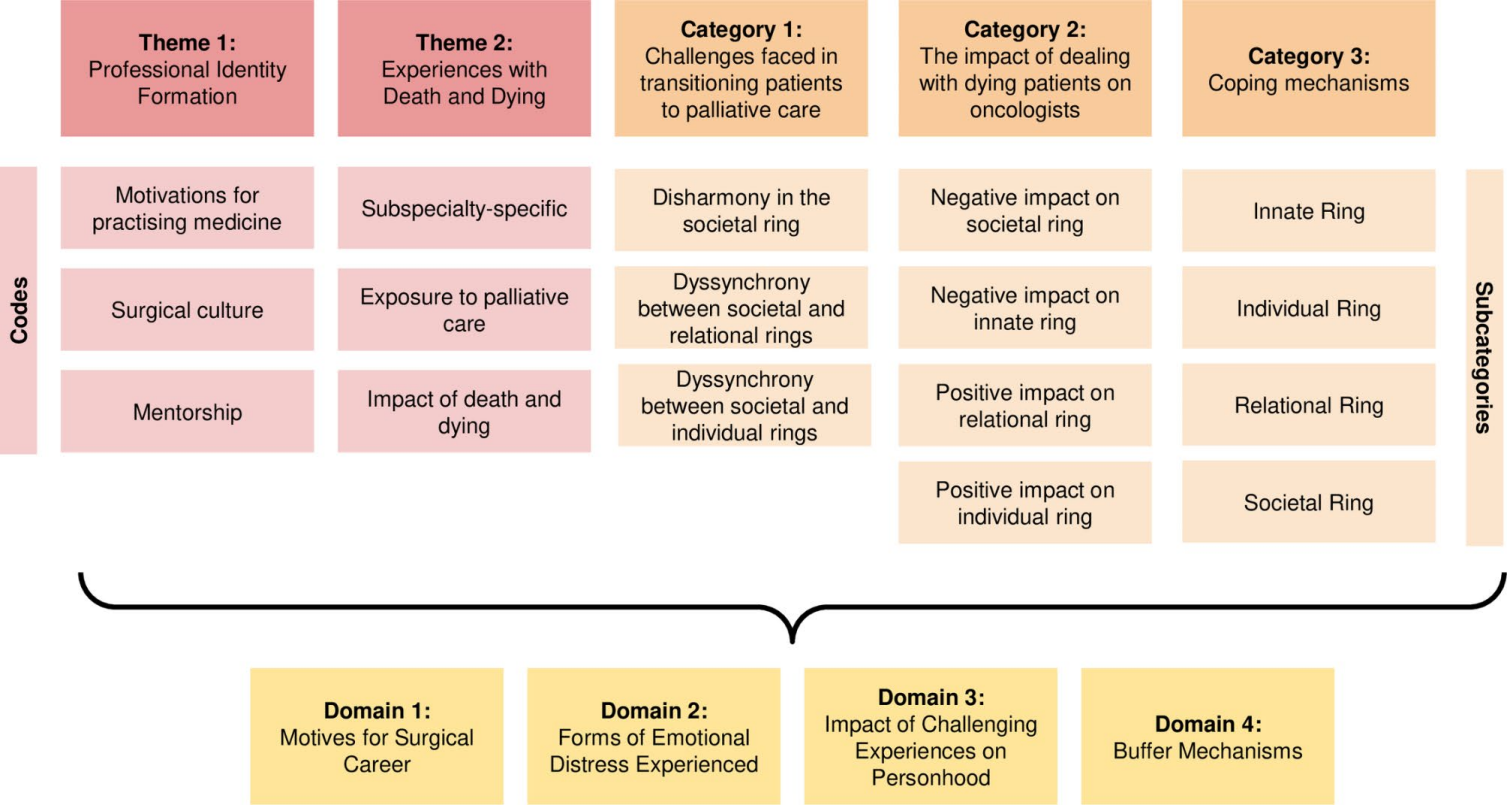
Coding tree

A coding tree was formulated during data analysis, depicted in Fig. 4.

Following the merging of complementary themes and categories in the Jigsaw Perspective, four key domains were identified: (1) motives for surgical career; (2) forms of emotional distress experienced; (3) impact of challenging experiences on personhood; and (4) buffer mechanisms.

### Domain 1. motives for surgical career

Surgeons were drawn to a career in medicine by a lifelong (S1, S10, S11) desire to help people (S1, S7, S8, S9, S11). A career in surgery facilitated a ‘definitive’ means of alleviating what ailed the patient (S1, S2, S5, S7, S8, S9, S10, S11):

In surgery, we can solve a lot of problems, right? There’s a hole in the stomach. You patch it. The patient gets better, right? If there’s a tumour, you take it out. Yes, the patient may need further treatment but that, you know, it is very effective treatment for many. (S8)

Surgery also promoted a modicum of control over proceedings (S6, S7, S8, S9, S10, S11):

You know there’s a problem and you can fix it. (S5)

I saw how effective it could be and maybe a few things are under your control. You could try and you could actually train to be a better surgeon and to effect better care. (S1)

This perception of control was inspired by mentors and role models (S1, S5, S8, S12):

In medical school, I think I had the honour of seeing good role models in general surgery. I enjoyed the nature of the job. (S8)

### Domain 2. forms of emotional distress experienced

Surgeons experienced a wide range of emotional distress in the forms of overlapping aspects of moral distress, vicarious trauma, compassion fatigue, secondary traumatic stress and burnout.

#### Moral distress

Being unable *“to act on core values and obligations”* [24] threatened the surgeons’ moral integrity and precipitated moral distress, as did *“distress resulting from one’s actions not achieving the desired outcome”* [63], or hinderances from effectively considering, balancing and executing a course of action [64, 65]:

What I would remember was that she was crying because she hadn’t even seen her baby yet because she was stuck in ICU or in high [dependency] and the baby, who was so pre-term, was also in the ICU… And that was… just in my mind since… It’s been like maybe 7–8 years. It’s a scene that I can still remember. Her face when she was crying, you know? Just like, like begging to see her baby. (S6)

#### Vicarious trauma

Witnessing emotional distress from the patients’ families and friends (S1, S3, S4, S6, S8, S10, S11) precipitated negative emotions amongst surgeons (S11):

My prof, yes, he definitely has thought about it, even like in the middle of the night. He will message me about her. So definitely, he can’t sleep, but he couldn’t sleep for that few months. Even when she passed on, he was thinking whether or not he should attend the wake and whether or not he should message the mother. (S6)

#### Compassion fatigue

Compassion fatigue, or physical and emotional exhaustion in caring for suffering patients and families, was noted by some surgeons:

Sometimes I still tend to, like, feel a bit overwhelmed with emotions and I’ll feel like I need to kind of, actually, I can’t even control my tears sometimes, if it gets too much. (S3)

I do know of colleagues who, you know, despite quite a few years of working, they might not have encountered as many deaths and… they are still quite shocked and emotionally affected by… an occurrence of death, whether it is expected or unexpected. (S9)

Further, compassion fatigue may be complicated by contextual considerations, such as interdisciplinary conflicts between teams. S2, for example, recounted compassion fatigue that arose when the intensivists suggested a conservative management plan concluding any escalation of care that ran contrary to the surgical team’s inputs. Balancing these conflicting perspectives was made harder by having to *“[keep] the family and patient up to date”* (S2) whilst dealing with *“seniors [debating whether] or not to give up or when to give up”* (S2).

#### Secondary traumatic stress

Secondary traumatic stress refers to distress faced when physicians are exposed to traumatic events associated with a blurring of boundaries between their personal and professional lives. S6 recounted how their senior pushed for aggressive treatment simply because they felt responsible for a misdiagnosis (S6):

This gynae patient was young. My own boss was very, very involved with this patient’s care. Like I think, he kind of felt responsible because what she had was… like a benign cyst and we found out actually it was misdiagnosed, like it was initially borderline and turned malignant… He attended all the meetings when it was about her, like the tumour board meetings, the theological things. And he got really very worked up, like he was scolding people for like not doing this. (S6)

#### Burnout

The toll of conflicting personal and professional demands contributed to burnout (S5, S6, S9). This was also complicated by a lack of a clear appreciation of burnout and inadequate self-awareness and self-care (S3, S5, S7, S8, S9, S11, S12):

I think like a lot of us go through the same experiences, right? You know, the same type of patients, the same volume of patients, but some experience burnout. Sometimes I think it’s like this coping mechanism. (S5)

### Domain 3. impact of challenging experiences on personhood

The exposure to trying situations bore ramifications for the surgeons and their personhood, as analysed through the lens of the RToP.

#### Innate ring

Caring for end-of-life patients led to some surgeons reflecting upon their conceptions of a ‘good life’ and ‘good death’. For some, a good life was associated with financial stability (S9), spending time with loved ones (S5, S7, S9, S11), independence (S5) and maximising quality of life (S5):

I think a good life would be… free of chronic illness and chronic pain for as long as it can be…You can spend time with your family and grow old relatively comfortably. That, to me, is a good life regardless of how wealthy you are or anything like that. And a good death probably, passing away in your sleep. (S11)

Surgeons further added that a good death would be one free of suffering, pain (S1, S4, S11) and unnecessary interventions, as well as being prepared for death (S3, S6, S8) in the company of loved ones (S2, S3, S6, S7, S8, S9, S10, S11, S12).

#### Individual ring

Surgeons acknowledged that their role was to *“[deal] with death”* (S10), rather than attempting to save every patient (S10, S11, S12). This fed the desire to ensure patients had the time to put their matters in order (S9) and not face life-prolonging interventions that brought little quality of life, symptom control and dignity (S3, S5, S7, S9).

#### Relational ring

On the relational front, the costs of caring saw surgeons initiate discussions on death and dying (S8) and introduce their loved ones (S1, S3, S7, S8) to advanced medical directives and care plans. Often, the impetus for these actions came after caring for patients with similar traits to their loved ones (S6, S8):

When I was a registrar, this case disrupted me because it’s a young kid that got knocked down by a car. It was a trauma case, OK? And usually, the institution doesn’t see paediatric cases. But it happened that the case was nearby, so they brought here first and the kid died and I think that was very disturbing to me because I have a kid about similar age. (S8)

#### Societal ring

Whilst exposure to death and dying varied between the different surgical subspecialties, all surgeons were exposed to the care of dying patients during their basic surgical training (S1, S2, S3, S6, S7, S8, S10). The impact of these early losses led some surgeons to pursue careers in breast and head and neck surgery often associated with lower mortality rates (S1, S2, S5, S8, S12). Clarity on the disease states also helped set expectations and define roles (S1, S4, S6, S8):

The last 15 years or so, my work has been mainly breast cancer. Obviously for surgery for breast cancer, we operate on those with curative intent. So, these are usually stage 1, 2 or even stage 3 breast cancers. Not by time. They’re stage 4 metastatic, rarely does the surgeon get involved. (S2)

Clear goals of care helped pave the way for discussions on the extent of care; the involvement of palliative care teams (S9, S11); and efforts to maximise quality of life, dignity and time spent with their loved ones (S4, S6, S9).

Nonetheless, surgeons struggled to balance their roles and responsibilities when patients made decisions that ran against their recommendations (S8) or belief systems (S3, S6, S8):

We know he’s not going to comply, he’s going to fix ideas and it kind of poses challenges in the treatment plan we put forward… But ultimately, one of the touchy points for him was the Do Not Resuscitate sort of status, because he was already a very advanced cancer stage… He wanted ECPR, he wanted his ICU and things like that… He actually deteriorated and a code blue was done and, ultimately, he passed away, and his wife actually thanked the team for doing the resuscitation, although medically, we struggled because we felt that it would have done more harm. (S3)

Surgeons also found it difficult to cope with the loss of patients who were young (S1, S3, S4, S6, S10, S11), admired (S1, S6), or had declined rapidly or unexpectedly (S1, S6, S10, S11):

One was a young father whose kids were, I think, all less than 10 years old. And so, when he came in, he passed away quite quickly. But we were quite close to him because he had been in and out of hospital. We’ve been treating him for quite long. (S10)

### Domain 4. buffer mechanisms

Patient care was limited by time constraints (S2, S5, S11), language barriers (S11) and failing to account for the patient’s particular situation and values (S9) that, together, impeded informed decision-making by patients and families (S2, S3, S9). To mitigate these challenges, surgeons developed several buffer mechanisms. These included developing good communication skills and body language (S1, S2, S3, S4, S6, S9, S12), building rapport (S2, S3), providing appropriate and timely information, breaking bad news (S4) in a personalised manner (S2, S4, S9) and offering condolences when patients passed (S4, S6, S9). Surgeons also reported that enhanced reflection, meaning-making (S9) and self-awareness of their practice aided with coping with distress (S1, S11):

I think that the ability to sit and think and also reflect and identify the inevitable responses to the encounters and then process it in a safe and meaningful way, it’s probably important. (S1)

I must say that even though in this particular case, you know, the worst outcome happened, but to me, it was a very invaluable learning experience. You know, learning from my colleagues on how to deal with all these complications, how to communicate with the family, how to get help from within the institution and of course learning how to deal with it. (S9)

However, whilst self-care was much vaunted, there remained a negative perception of psychological care and counselling:

I think there’s still a feeling that if you seek help within the institution that somehow it will be seen as a negative for you that you’re having to do that. (S11)

Seeking the help of a psychiatrist was similarly mired in negative connotations:

To recognise burnout himself and then seek help sounds very American to me. I’m not saying there’s no problem but putting up the front… is a bit more like the British stiff upper lip. Like Americans are depressed, everyone seeing a psychiatrist in America. (S2)

I don’t think people talk about it [the impact of death and dying on their emotional or mental well-being] very much, you know, mental well-being in general. Nobody started talking about it until the last couple of years… I mean, our culture is just one where we are not that more open to sharing. (S10)

## Discussion

In mapping the experiences of surgeons in Singapore as they care for end-of-life patients and their families and interact with multi-disciplinary teams, this SEBA-guided study reveals a unique intermix of context- and speciality-dependent, practice-culture-led considerations and individual influences. To answer the primary research question, *‘What is known about the costs of caring on surgeons in Singapore?’*, it was found that surgeons are not immune to the costs of caring, in itself a blend of compassion fatigue, secondary traumatic stress, vicarious trauma, moral distress and burnout, echoing previous studies [28, 66]. Similarly, the impact on surgeons and coping mechanisms employed can be structured according to the various rings in the RToP, illustrating the broad impact that caring for dying patients has on the various aspects of the individual’s sense of self, including their professional identity.

What is ever more pronounced in this study is the influence of surgical culture in coping with the costs of caring compared to previous studies amongst medical specialties [28, 66, 67]. This influence is evident as early as the decision to pursue a career in surgery, as illustrated in Domain 1 of the results. Surgeons aspire to solve problems, cure and/or correct life-altering issues definitively as part of an underlying desire to help others. Surgery offers the clinicians an element of control in this process where ‘losing a patient’ remains the antithesis of a general surgical ethos. The same may be said in accepting a palliative stance in a field still steeped in a ‘never give up’ culture. Some surgeons turn to careers where patient mortality is low and less prone to sudden deteriorations, where clearer disease trajectories allow for the alignment of expectations and the establishment of the goals and extent of care. However, eventually shifting from a ‘curative imperative’ to one focused on comfort measures and respecting the patient’s overall goals of care can be challenging. This ‘discomfort’ accumulates. Evidence of frequent exposure to patient loss, traumatic deaths, grieving families and morally distressed teammates extend the costs of caring, as described in Domains 2 and 3.

We posit that these costs of caring are buffered through the *internal* compass, described as rooted belief systems that guide how surgeons think, feel and act as professionals—honed by growing experience, insights, reflections and integration of the local culture. The *internal compass* directs how surgeons perceive challenges to their belief systems, adjudge and prioritise action to address them whilst balancing the wider considerations in adapting their self-identity. Built on the belief systems rooted within the individual’s RToP, the notion of the *internal compass* shepherds decisioning throughout the KPM’s processes. We believe the *internal compass* may be a meaningful and sustainable way to navigate clinically and ethically complex situations, thereby bridging understanding in how PIF influences practice and conduct whilst serving as a barrier against costs of caring that, if left unattended, may culminate in burnout [68].

Whilst it is clear that more research is required to evaluate the posits drawn from this small study, we believe there is sufficient data to advocate for holistic, consistent and personalised support of local surgeons. Drawing on our earlier reviews, this support could take the shape of mentorship programmes and longitudinal multimodal assessment tools, such as reflective portfolios and diaries. These ought to be supported by guided debriefs and peer-mentoring. Further studies may include exploring the effectiveness of such interventions in mitigating the costs of caring and examining the long-term impact of these costs on surgeons’ personhood and careers.

### Limitations

This study was only carried out with senior surgeons of a single tertiary cancer centre in Singapore, potentially limiting its generalisability. Much of the data collected is prone to recall bias and compounded by the lack of longitudinal studies. Further, the study team included a general surgeon as the first author, alongside medical students and junior and senior physicians, who practice within a similar sociocultural context. This might have introduced biases within the analysis, including confirmation bias, where preconceived notions of surgeons’ experiences influenced interpretation, and social desirability bias, where participants minimised negative accounts due to a plausible dependent relationship with the lead author. Oversight by an expert team, independent thematic and content analyses by separate researchers, as well as recruiting interviewers without dependent relationships with the participants, aimed to minimise these effects. Additionally, as some eligible surgeons did not respond to the study invitation, the results might be skewed, potentially overrepresenting either positive or negative accounts based on the participants’ varying levels of motivations to share their experiences.

## Conclusion

The costs of caring for the dying and contending with grieving families and distressed team members evolve with meaning-making, reflection and growing experience, competencies and insights. Assessing these effects and their influence upon practice will be the focus of our coming studies as we look forward to greater understanding and support for surgeons, with the hope that the findings will guide the support of other healthcare professionals.

## Electronic supplementary material

Below is the link to the electronic supplementary material.

**Supplementary Material 1**: **Additional File 1**. COREQ Checklist

**Supplementary Material 2**: **Additional File 2**. Interview Guide

## Data Availability

The authors confirm that the data supporting the findings of this study are available within the article and its supplementary materials.

## Abbreviations

PTSD: Post-traumatic stress disorder
SEBA: Systematic evidence based approach
PIF: Professional identity formation
KPM: Krishna-pisupati model for professional identity formation
COREQ: Consolidated criteria for reporting qualitative studies
