# Supplementary material for: The impact of death and dying on surgeons in a tertiary cancer centre in Singapore
Source: BMC Surg. 2025 May 5;25:196. doi: 10.1186/s12893-025-02881-1 (PMC12051349; doi:10.1186/s12893-025-02881-1)
Supplement: Supplementary file 2 — Supplementary Material 2: Additional File 2. Interview Guide [file 12893_2025_2881_MOESM2_ESM.docx]

**Additional File 2. Interview Guide**

| **Introduction** | - In this study, we hope to understand the impact of caring for dying patients on you. Specifically, we are interested in how these experiences impact your personhood, or ‘what makes you, you’. - We believe that individual concepts of personhood are determined by 4 domains which relate to your religious/existential beliefs your ties and roles within society, your relationships with your family and those that matter with you and your conscious function which is your ability to think, talk and act autonomously. We hope to understand how your experiences have affected these elements of ‘what makes you, you’. - This interview will be audio recorded and anonymized for analysis. The audio recording will be deleted after the study is complete. Do I have permission to record and begin the interview? |
| --- | --- |
| **Topic Areas** | **Questions** |
| **Demographic Questions** | ***To start off this interview, I would like to understand more about your background in medicine.***   1. Could you share with me what drew you to medicine/surgery?  - Were there any personal values or beliefs that attracted you to the field?  1. How long have you been in your field? 2. Because this study surrounds dying patients and end-of-life care, could you give a rough percentage as to the number of patients that you see that would fall under this category? 3. On this topic of end-of-life care, I would like to know, how do you view life and death?    - For e.g., could you share with us what you think makes a “good life” or “good death”? 4. Has this concept of life and death always been there, or has it changed as you came into this practice and worked with patients? / Has your experience in healthcare shaped what you believe is a good life or death? 5. Do you think these beliefs influence your decision-making and the actions that you take in your personal and professional life? If so, how? 6. What are the skills you possess that you believe are important to have as a physician caring for patients?  - Soft skills*? E.g. communication, empathy*  1. Are these skills honed over time in your years of practice or have they always been there? 2. As a healthcare practitioner, what roles and responsibilities do you have towards your patients? |
|  | ***Thank you for sharing. Maybe we can move on to a memorable case now.***   1. Could you share with us a **memorable case** that has left a lasting impact on you? Maybe something that has rocked your confidence or beliefs, huge conflicts, lessons learned, anything that comes to mind?  \| **MEMORABLE CASE**  **RToP PROMPTS:**   1. **Innate:** How did it affect your religious/spiritual beliefs/perspective of a good life and/or death? 2. **Individual:** How did this case affect the way you see yourself/what you value in life? 3. **Relational**: How does this case affect the way you think about your family/ those who matter to you?  - *Some doctors we interviewed have said that caring for dying patients makes them distance themselves from their loved ones for fear of loss while others choose to spend more time with family.*  1. **Societal:** Did this case change or affirm any specific role or responsibilities you have as a physician?  - Did you wish or feel like you could have done more for the patient? - Do you speak to patients and their families regularly in your day-to-day? - What kind of fears pop up? - Do these conversations influence you in any way?   **FOLLOW-UP PROMPTS:**   1. Why was this case memorable? 2. What were the most challenging aspects of this encounter? *e.g., spiritual, emotional, psychosocial* 3. How have you adapted or changed following the case? 4. What helped you find an answer and balance? (Who or what helped you in achieving resolution?)  - Is this something you had to learn across the years, or did it come to you naturally?  1. What was your source of support during difficult encounters like these? Do you seek support from colleagues/mentors or family, friends etc.?  - Why do you find yourself seeking support from __ and not others?   **OTHER PROMPTS:**   1. What were the patient’s needs? 2. What were the loved ones’ needs? 3. How did you respond to their needs? \| \| --- \|  1. More generally, do you think that caring for patients brings specific complications that affect your emotional, psychological or spiritual well-being? Possible prompts:  - Has interacting with suffering/dying patients changed any of your own spiritual or religious beliefs, if you have any? - For some physicians and nurses, encountering death often either instils a certain fear about death or hopefulness about death. Have your thoughts on death changed?  1. How does this affect the way you think and view yourself as a healthcare professional?  - Some physicians expressed feelings of helplessness in not being able to fully fulfil their roles whilst others come to understand & appreciate the limitations of their roles.  1. How do you deal with these feelings? 2. In your years of working closely with suffering/dying patients and their families, did you ever find yourself getting too emotionally invested in a patient? 3. Did you ever find yourself bringing work/negative emotions back home? Did it affect your own personal life? 4. Through your years working with suffering/dying patients and their families, were there any lessons that you have learned along the way? E.g. professional care delivery, work-life balance, new outlooks on life, maintaining professional boundaries etc. |
| **Conflicts and Moral Distress** | ***Thank you for sharing. I understand that caring for patients comes with different, often times conflicting, expectations, beliefs, and opinions.***   1. For you, has there ever been some conflict in what you believed in versus what was expected of you as a physician?  - E.g., some doctors we had interviewed struggled with caring for suffering patients as they were questioning whether they were prolonging the dying processes rather than prolonging a good quality of life for the patient.  1. Moral distress arises in situations where your personal moral/ethical values are in conflict with the needs, preferences or decisions of the patient, their family, the medical team, or the larger healthcare system. **Have you experienced instances of moral distress in your line of work?**  - For more clarity or specification: Were there any instances where you were put into a very difficult position making clinical decisions, because of patient or collegial opinions? - **[ON RELIGION]** Have you ever encountered patients with very strong views that were different from yours?   - How do you come to reconcile these differences?   - Do you think it’s important for the healthcare team to be multi-religious & multicultural to help patients navigate through this?  1. What was your source of support during difficult encounters like these? Do you seek support from colleagues/mentors or family, friends etc.?  - Why do you find yourself seeking support from __ and not others?  1. Are you more sensitive to recognize moral distress over time/ with experience?  - If so, how do you now detect moral distress?  1. How has experience changed your judgement/ assessment of moral distress? 2. Are you more willing to address moral distress? 3. If so, how do you address moral distress?  - Do you have other coping mechanisms in place?  1. Do you believe that your institution has provided effective support, be it in the form of education or psycho-emotional support, in addressing distress in caring for suffering/dying patients?  - What changes would you like to see in the institution, if any? |
| **[OPTIONAL]**  **Conflict & Dyssynchrony in Suffering, Euthanasia and Death & Dying** | **Possible prompts:**   - Have you witnessed healthcare providers giving **“false hope”** to a patient or family? - Have you participated in care that compromised the **dignity** of the patient? - Have you witnessed a **violation of a standard of practice or a code of ethics** and not felt sufficiently supported to report the violation? - Have you witnessed compromised patient care due to **lack of resources, equipment, bed capacity or administrative support**? - Have you witnessed **stigmatizing social circumstances** or conditions (e.g. alcoholism, drug abuse, homelessness, or obesity) leading to less than optimal care being provided? |
